# Editorial: Transforming health and social education to include a greater focus on public health education in the curriculum

**DOI:** 10.3389/fpubh.2023.1306124

**Published:** 2023-11-24

**Authors:** Sharon Brownie, Louise Ackers, Georgina Murphy, Constance Shumba

**Affiliations:** ^1^School of Health Sciences, Swinburne University of Technology, Hawthorn, VIC, Australia; ^2^School of Medicine and Dentistry, Griffith University, Gold Coast, QLD, Australia; ^3^Centre for Health and Social Practice, Te Pukenga: Wintec, Hamilton, New Zealand; ^4^Global Social Justice, University of Salford, Salford, United Kingdom; ^5^Bill & Linda Gates Foundation, Seattle, WA, United States; ^6^School of Nursing and Midwifery, Aga Khan University, Nairobi, Kenya

**Keywords:** public health, curriculum, health and social education, diversity, health priorities

The Research Topic “*Transforming health and social education to include a greater focus on public health education in the curriculum”* has published 12 articles with 73 contributing authors from at least 11 countries demonstrating global interest in reshaping curricula across the future healthcare workforce. The articles depict diverse public health priorities for health and social education across a variety of contexts. The diversity of the Research Topic is indicative of the rapidly changing healthcare landscape in which we see new treatment discoveries; changing societal values and perspectives; technological innovation; increased expectations of personalized healthcare; and unresolved inequity despite the advances in healthcare discovery and innovation. The Research Topic adds to a growing body of literature calling for, update and change in public health education (1–4). Perspectives and interventions within the manuscripts provide examples of the direction that health and social education might take in the future in areas reinforcing the need for curricula change and preparation of new cadres of health professionals.

The changing public health priorities call for parallel changes in the **educational preparation** of the health workforce (5). Community Health Workers (CHWs) play a crucial role in public health by providing preventive and promotive health services, taking services closer to their communities, and increasing access to facility-based health services. Rogers et al. explored education, literacy, experience, training, and gender as potential predictors of Community Health Workers' (CHWs) performance in Migori County, Kenya. Results demonstrate that educational intervention has the capacity to contribute to increased knowledge among CHWs and a significant predictor of immunization rates and antenatal care completion rates among the clients reached by CHWs. The framework developed by Armstrong et al., provides a forward-thinking model for the development of population health competencies within the future medical workforce with the aim of ensuring greater understanding and commitment to population health priorities. Nursing curricula provide an important entry point to address community level MCH concerns and bolster MCH outcomes. Isangula et al. co-designed an intervention package (prototype) to improve nurse–client relationships using a human-centered design approach with maternal and child health nurses and clients in rural Tanzania. One of the key interventions increased hours within the nursing education curriculum to focus on communication skills, customer care and patient-centered care, to improve nurse-client relationships—particularly important skills in primary care settings. Effective interprofessional strategies are needed to better address burgeoning public health issues such as T2DM, however, the acquisition of interprofessional competencies (IPC's) does not occur by chance. IPC's must be actively taught and assessed. Brownie et al. provide a collated guide of the IPE assessment tools available for teaching faculty wishing to support IP competency development for the future workforce. Aging of the population. Changes are needed across the full workforce with specific attention to all cadres of the health care team including allied health professionals (Dalton et al.) whose roles are increasing important.

As much as 50% of health professional **education** occurs **in clinical settings**. In order to effect real change, enhancements within written curricula must flow from class-based settings class to clinical education context where students are able to see and reflect upon grass-roots public health challenges including both chronic and emergency settings (Jie et al.) The scoping review by Sampath et al. investigated the prospect for curriculum enhancement through the role of Student run free clinics (SRFCs) and their effectiveness in the prevention and management of type 2 diabetes mellitus (T2DM) among indigenous older adults. The review found that SRFCs are particularly beneficial in providing high quality and effective T2DM management for underserved populations with no health insurance and of lower socio-economic status. Female cervical cancer deaths in sub-Saharan Africa, continue to be a major public health issue despite being both preventable and curable if detected and treated early. The Community Case Study by Auma et al. presents a multi-pronged, technology informed, point-of-care screening intervention using a series of action-research cycles to scale-up screening and treatment of cervical cancer through a community-based model in Uganda. The cycles included device procurement, capability-enhancement, the use of Geographic Information Systems to guide awareness-raising and service integration with HIV care. In accordance with the Ministry of Health guidelines in Uganda, low-cost screening is done using Visual Inspection with Acetic Acid and treatment of early dysplasia (cervical intraepithelial neoplasia) using cryotherapy. The action-research cycles were progressively integrated into a comprehensive, task-shifted, point-of-care, prevention program in a community-based public health facility. The authors found that task-shifting responsibility to Community Health Workers and the application of Geographic Information Systems to strategically guide health awareness-raising and the deployment of medical devices was effective in supporting respectful and sustainable point-of-care screen-and-treat services. Further, integration with HIV services increases reach among those at the highest risk of cervical cancer. The integration of this with public HIV services demonstrates the ability to engage hard-to-reach “key populations” at greatest risk of cervical cancer. The model presents opportunities for policy transfer to other areas of health promotion and prevention with important lessons for international health partnership engagement. Findings also demonstrate the impact of external influences and the Results Based Financing approach, adopted by many foreign NGOs. The works of Auma et al., Case et al. and Sun et al. demonstrate the rapid expansion of technological innovation with which curricula transformation must keep pace.

Societal values, beliefs and mores are continuously changing with increased commitment to more inclusive and culturally attuned care provision with significant opportunity for curriculum transformation in these areas Promoting inclusiveness and the provision of culturally competent healthcare among Lesbian, Gay, Bi-sexual, Transgender, Questioning, Intersex, and Asexual (LGBTQIA+) patients is an area of growing public health importance to counter persistent discrimination in the provider-client encounters. Prasad et al. assessed the educational impact of an active learning session that was specifically designed to enhance LGBTQIA+ cultural competency awareness using an interprofessional setting. Students in the study identified self-reflection processes as being crucial in addressing implicit biases regarding LGBTQIA+ individuals. The authors pointed to how useful expanded culturally competent interprofessional collaboration through education and awareness could be in improving healthcare for LGBTQIA+ patients. Health delivery transcends national boundaries with practitioners working across a variety of settings. Fostering cross-cultural communication skills by leveraging platforms and tools that enhance social connections and communication. Ahmad et al. and Case et al. both implemented virtual programs aimed at developing students' intercultural competency by engaging students from Australia and India and the US and Egypt respectively. Both used a peer-to-peer approach with the Australia- India interactions being synchronous while the US-Egypt study used both text and synchronous Zoom medium. Both studies provide important insights on the role that virtual platforms can be utilized in public health to support intercultural learning among students as peers.

The Research Topic provides useful examples in class and clinical education settings illustrative of the breadth of the health workforce—HCW's doctors, nurses, and allied health personnel. The diversity of the Research Topic is indicative of the rapidly changing healthcare landscape in which we see new treatment discoveries; changing societal values and perspectives; technological innovation; expectations of personalized healthcare; and embedded inequity despite the advances in healthcare discovery and innovation. In short, nothing stands still, and each decade heralds a very different practice landscape into which graduate health professionals emerge. The diversity of studies points to the need to continuously examine and renew the public health curriculum.

## Author contributions

SB: Conceptualization, Methodology, Project administration, Supervision, Writing—original draft. LA: Conceptualization, Writing—review & editing. GM: Conceptualization, Writing—original draft. CS: Conceptualization, Methodology, Writing—original draft.
